# Reversible 3D-2D structural phase transition and giant electronic modulation in nonequilibrium alloy semiconductor, lead-tin-selenide

**DOI:** 10.1126/sciadv.abf2725

**Published:** 2021-03-19

**Authors:** Takayoshi Katase, Yudai Takahashi, Xinyi He, Terumasa Tadano, Keisuke Ide, Hideto Yoshida, Shiro Kawachi, Jun-ichi Yamaura, Masato Sasase, Hidenori Hiramatsu, Hideo Hosono, Toshio Kamiya

**Affiliations:** 1 Laboratory for Materials and Structures, Institute of Innovative Research, Tokyo Institute of Technology, 4259 Nagatsuta, Midori, Yokohama 226-8503, Japan.; 2 National Institute for Materials Science, Sengen, Tsukuba 305-0047, Japan.; 3 The Institute of Scientific and Industrial Research, Osaka University, 8-1 Mihogaoka, Ibaraki, Osaka 567-0047, Japan.; 4 Materials Research Center for Element Strategy, Tokyo Institute of Technology, 4259 Nagatsuta, Midori, Yokohama 226-8503, Japan.

## Abstract

Material properties depend largely on the dimensionality of the crystal structures and the associated electronic structures. If the crystal-structure dimensionality can be switched reversibly in the same material, then a drastic property change may be controllable. Here, we propose a design route for a direct three-dimensional (3D) to 2D structural phase transition, demonstrating an example in (Pb_1−*x*_Sn*_x_*)Se alloy system, where Pb^2+^ and Sn^2+^ have similar *n*s^2^ pseudo-closed shell configurations, but the former stabilizes the 3D rock-salt-type structure while the latter a 2D layered structure. However, this system has no direct phase boundary between these crystal structures under thermal equilibrium. We succeeded in inducing the direct 3D-2D structural phase transition in (Pb_1−*x*_Sn*_x_*)Se alloy epitaxial films by using a nonequilibrium growth technique. Reversible giant electronic property change was attained at *x* ~ 0.5 originating in the abrupt band structure switch from gapless Dirac-like state to semiconducting state.

## Teaser

3D-2D structural phase transition is artificially induced to invoke giant electronic modulation in nonequilibrium (Pb_1−*x*_Sn*_x_*)Se.

## INTRODUCTION

Pseudo-closed electronic configurations of *n*s^2^ ions such as Sn^2+^, Pb^2+^, and Bi^3+^ have similar chemical behaviors but realize a variety of crystal structures including three-dimensional (3D) structure, 2D layered structure, 1D channel structure, and 0D structure, originating from the competition among various types of chemical bonds such as the ionic bonds, the covalent bonds, and the *n*s^2^ lone-pair electrons (*1*–*5*). A particular interest is in a direct phase transition between largely different crystal structures, e.g., between a 2D layered structure and a 3D crystal structure, as such system is expected to exhibit a giant property modulation and will be applied to optoelectronic devices if the structural phase transition is controlled by external stimuli such as temperature and electric field. However, such direct structural phase transition has not been reported, to our knowledge; only pressure-induced transitions between 2D and 3D structures are reported (*6*, *7*).

**Fig. 1:**
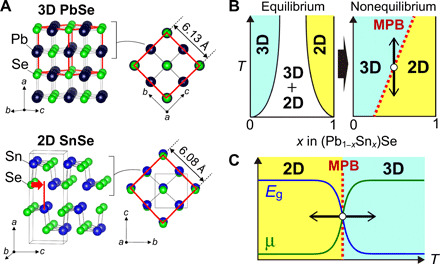
Strategy for reversible 3D-2D structural phase transition in (Pb_1−*x*_Sn*_x_*)Se alloy. (**A**) Crystal structures of 3D RS-type cubic PbSe (top) and 2D GeS-type orthorhombic SnSe (bottom). The left and the right panels show the side view and the top view of the crystal structures, respectively. The red cube and the red squares indicate the RS-type conventional unit cell and the $\sqrt{2}\times \sqrt{2}$ supercell unit of the GeS-type structure that corresponds to the RS-type conventional unit cell, respectively. If adjacent layers in SnSe are shifted by (0, 0, 0.38) along the *c* axis with each other, then the GeS-type structure is transformed to the RS-type structure, as indicated by the red arrow in the left bottom panel. (**B**) (Left) Typical composition (*x*) versus temperature (*T*) phase diagram for (Pb_1−*x*_Sn*_x_*)Se alloy under equilibrium and (right) that under nonequilibrium state. If the morphotropic phase boundary (MPB) is formed at a critical *x* like in the right panel, then direct 3D-2D structural phase transition can be induced by temperature (*T*), as indicated by the vertical arrows. (**C**) Schematic image of the giant modulation for carrier mobility (μ) and bandgap (*E*_g_) by the 3D-2D structural phase transition at MPB.

In this work, we focused on a chalcogenide semiconductor alloy system of the rock salt (RS)–type PbSe composed of Pb^2+^ with the 6s^2^ configuration and the layered GeS-type SnSe composed of Sn^2+^ with the 5s^2^ configuration. Figure 1A compares these crystal structures. Pb^2+^ stabilizes a 2D layered structure in oxides as seen in PbO (*8*), while it stabilizes the RS-type structure in Pb*Ch* chalcogenides (*Ch* = S, Se, and Te) with the high-symmetry cubic structure (space group, *Fm*$\bar{3}$*m*) formed by a 3D network of edge-shared (Pb-*Ch*_6_) octahedra (*9*). The high coordination number polyhedra and the short Pb-Pb distance form a large band dispersion (i.e., small carrier effective mass) (*10*, *11*) and a small bandgap (*E*_g_) of ~0.3 eV (*12*, *13*) in PbSe. In particular, the RS-type (Pb_1−*x*_Sn*_x_*)Se has gathered much attention as a topological crystalline insulator (*14*, *15*). That is, the substitution for Pb with Sn in the RS-type PbSe reduces the bandgap and lastly produces a gapless Dirac-like state, where the valence band and the conduction band in (Pb_1−*x*_Sn*_x_*)Se approach each other and lastly invert above the critical *x* = ~0.3 at room temperature (RT) (*16*).

On the other hand, GeS-type SnSe with Sn^2+^ 5s^2^ has the anisotropic layered structure (space group, *Pnma*) composed of the alternately stacked SnSe layers with threefold coordinated Sn and Se along the *a* axis (*17*). Note that SnSe has band dispersions perpendicular to the SnSe layer (*18*), indicating that SnSe is not 2D in electronic structure. On the other hand, SnSe single crystals have 10 times higher conductivity in the SnSe layer than that perpendicular to the SnSe layer. Therefore, SnSe shows highly anisotropic carrier transport, reflecting the 2D crystal structure. The low symmetric structure and lower coordination number of Sn reduce the band dispersion (i.e., larger carrier effective mass) and increase the bandgap up to 0.9 to 1.0 eV (*19*–*23*). Actually, the reported mobility [150 to 200 cm^2^/(V·s) for hole] of SnSe single crystals is far smaller than that of PbSe single crystals [830 to 930 cm^2^/(V·s) for hole and 1200 to 1300 cm^2^/(V·s) for electron] (*19*, *20*, *24*, *25*). The GeS-type structure can be regarded as a distorted RS-type structure, as seen in the top view of these crystal structures (the right panels of Fig. 1A), where the distorted face-centered structure with $\sqrt{2}\times \sqrt{2}$ supercell unit is found in the SnSe one-molecular layer (the red square in the right bottom panel) although adjacent layers are shifted with each other along the *c* direction. If the adjacent molecular layers are shifted by (0, 0, 0.38) along the *c* axis as indicated by the red arrow in the left bottom panel of Fig. 1A, then the GeS-type structure is transformed to the RS-type structure (the left top panel), and it is of diffusionless transition (*26*, *27*). Therefore, if these pseudo-isostructures can be switched by external stresses such as temperature and electric field, then it would lead to a giant functional phase transition, such as large carrier mobility change and topological state transition, enhanced by the distinct electronic structure changes.

The alloying PbSe and SnSe would manipulate the drastic transition in structure, and such (Pb_1−*x*_Sn*_x_*)Se alloy should induce strong frustration around phase boundaries. However, as illustrated in the left panel of Fig. 1B, there is no direct phase boundary between the single-phase regions of the 3D PbSe and the 2D SnSe phases under thermal equilibrium; i.e., the solubility limit in the 3D (Pb_1−*x*_Sn*_x_*)Se is limited to *x* = 0 to 0.37 (*14*) and that in the 2D one to *x* = 0.80 to 1.0 (*28*), and a mixed-phase region lies between them.

To overcome this issue, we developed a nonequilibrium film growth technique combining reactive solid-phase epitaxy (R-SPE) and thermal rapid quenching process (*29*). In this method, the epitaxial growth of an RS-type (Pb_1−*x*_Sn*_x_*)Se film is induced by a solid-state reaction of a SnSe film deposited on an epitaxially grown PbSe template layer, where RS-type (Pb_1−*x*_Sn*_x_*)Se is stabilized at the high-temperature reaction process (*30*, *31*). The subsequent rapid quenching to RT is effective to freeze the high-temperature RS-type (Pb_1−*x*_Sn*_x_*)Se phase down to RT, forming the nonequilibrium phase. As a result, we have succeeded in extending the RS-type (Pb_1−*x*_Sn*_x_*)Se composition region up to *x* = 0.5 from the already reported *x* = 0.37 (*14*). We expect that an artificial morphotropic phase boundary (MPB) can be found in the nonequilibrium (Pb_1−*x*_Sn*_x_*)Se alloy film, which should induce the direct phase transition from the 3D to the 2D crystal structures (right panel of Fig. 1B) and large modulation of electronic properties, i.e., the bandgap and carrier mobility, by temperature (Fig. 1C).

Here, we report the artificially induced 3D-2D direct structural phase transition and electronic property modulation in the nonequilibrium (Pb_1−*x*_Sn*_x_*)Se alloy. It was found that the direct 3D-2D structural phase transition was induced around the critical composition *x* = 0.5. The 3D-2D structure change was reversibly controlled by temperature, and the giant modulation in electron mobility at three orders of magnitude was observed because of the abrupt band structure change from a gapless Dirac-like state to a semiconducting state.

## RESULTS

### Synthesis of (Pb_1−*x*_Sn*_x_*)Se alloy films

**Fig. 2:**
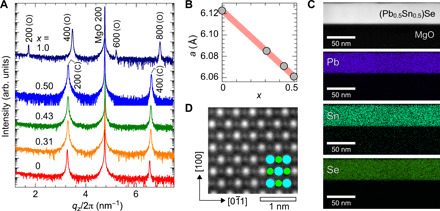
Crystal structures of (Pb_1−*x*_Sn*_x_*)Se alloy films on MgO (100) substrates. (**A**) Out-of-plane XRD patterns for *x* = 0 to 1.0. Crystalline phases [denoted as (C) for cubic RS type and (O) for orthorhombic GeS type] and diffraction indices are given for the corresponding diffraction peaks. (**B**) Lattice parameter as a function of *x*. (**C**) Cross-sectional STEM image and EDS mappings of Pb, Sn, and Se for (Pb_0.5_Sn_0.5_)Se film on MgO substrate. (**D**) Atomic-resolution cross-sectional HAADF-STEM image for (Pb_0.5_Sn_0.5_)Se film region. The atomic structure image of the RS-type phase is superimposed in the HAADF-STEM image, where the blue and green spheres indicate Pb/Sn and Se, respectively.

We first synthesized the (Pb_1−*x*_Sn*_x_*)Se epitaxial films on MgO (100) substrates by our nonequilibrium two-step process combining the R-SPE method and subsequent rapid quenching from 600°C to RT into iced water (see fig. S1). *x* was controlled up to 0.5 by changing the film thicknesses of the PbSe template layer and the SnSe top layer, i.e., by the thickness ratio of 1 − *x*:*x* for PbSe:SnSe. Figure 2A shows out-of-plane x-ray diffraction (XRD) patterns at RT, where the XRD patterns of cubic PbSe (*x* = 0) and orthorhombic SnSe (*x* = 1) epitaxial films are shown for comparison. The diffraction peaks for the (Pb_1−*x*_Sn*_x_*)Se films with *x* ≤ 0.5 are assigned to the *h*00 diffractions (*h* = 2 and 4) of the cubic RS-type phase along with the 200 diffraction of the MgO substrate. For pure SnSe film, the *h*00 diffractions (*h* = 2, 4, 6, and 8) originate from the layered stacking structure in the GeS-type phase with the *a*-axis orientation perpendicular to the substrate surface. These results substantiate that all the (Pb_1−*x*_Sn*_x_*)Se alloy films up to *x* = 0.5 maintain the cubic RS-type structure at RT. The detailed XRD analysis on epitaxial structure for (Pb_0.5_Sn_0.5_)Se film is shown in figs. S2 and S3. The *h*00 diffraction peaks of the RS-type (Pb_1−*x*_Sn*_x_*)Se films shift systematically to higher angles from the diffraction peak of the pure PbSe film as *x* increases. Figure 2B shows the evolution of the lattice parameter for the (Pb_1−*x*_Sn*_x_*)Se films as a function of *x*. The systematic and linear shrinkage of the lattice parameter is observed (the largest Δ*a*/*a* was ~−1.01% for *x* = 0.5), being consistent with the model that the smaller Sn^2+^ ion substitutes the Pb^2+^ site in the epitaxial films. These results indicate that we succeeded in stabilizing the high-temperature nonequilibrium RS-type (Pb_1−*x*_Sn*_x_*)Se and continuously controlling *x* up to 0.5 in the epitaxial films.

The microscopic structure analysis of the (Pb_0.5_Sn_0.5_)Se film was carried out by high-angle annular dark-field scanning transmission electron microscopy (HAADF-STEM). Figure 2C shows the cross-sectional bright-field STEM image and energy-dispersive x-ray spectroscopy (EDS) mappings. The chemical composition mappings of Pb, Sn, and Se clearly support the uniformity in the whole region of the film. Figure 2D shows the atomic structure of the (Pb_0.5_Sn_0.5_)Se film, which clearly visualizes the cubic lattice of RS-type phase. The interface structure between the film and the MgO substrate is shown in fig. S4. The atomic structure of the cubic lattice is seen from the bulk region to the interface, and the pseudo-coherent interface is observed (*32*), implying that the films grew heteroepitaxially on MgO (100) single crystals with the relaxed crystalline lattice.

### Electronic transition of (Pb_1−*x*_Sn*_x_*)Se alloy

**Fig. 3:**
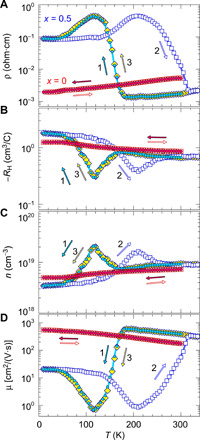
Electronic transition of (Pb_1−*x*_Sn*_x_*)Se alloy films. (**A** to **D**) Temperature dependences of (A) resistivity (ρ), (B) Hall coefficient (*R*_H_), (C) carrier concentration [*n =* 1/(*eR*_H_)], and (D) Hall mobility [μ = 1/(*en*ρ)] for PbSe (*x* = 0) and (Pb_0.5_Sn_0.5_)Se (*x* = 0.5) epitaxial films. For PbSe film, the measurement started from a cooling process (the closed red symbols) and then switched to a heating process (the open red symbols). For (Pb_0.5_Sn_0.5_)Se film, the measurement started from a cooling process (1) and switched to a heating process (2) and then returned to a cooling process (3).

**Fig. 4:**
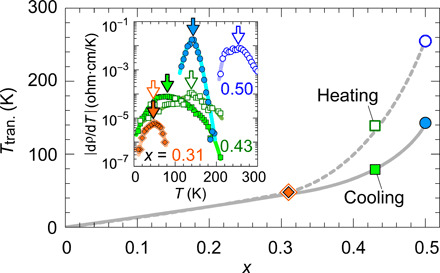
Electronic transition temperature of (Pb_1−*x*_Sn*_x_*)Se alloy films. ρ transition temperature (*T*_tran._) as a function of *x* under cooling process (closed symbols) and heating process (open symbols). *T*_tran._ are determined as the peak positions (indicated by arrows) of the dρ/d*T*-*T* curves around the phase transition in the inset.

Figure 3 (A to D) shows the temperature (*T*) dependences of (A) resistivity ρ, (B) Hall coefficient *R*_H_, (C) carrier concentration calculated by *n* = 1/(*e*|*R*_H_|), and (D) Hall mobility calculated by μ = 1/(*en*ρ) for pure PbSe film (red symbols) and (Pb_0.5_Sn_0.5_)Se film (blue symbols). Those for the other (Pb_1−*x*_Sn*_x_*)Se films are given in fig. S5. The measurement for (Pb_0.5_Sn_0.5_)Se film started from a cooling process from *T* = 300 to 10 K and switched to a heating process from 10 to 335 K and then returned to a cooling process from 335 to 10 K. All the (Pb_1−*x*_Sn*_x_*)Se films except for pure SnSe (*x* = 1.0) show n-type conductivity, which is confirmed by the negative *R*_H_ and Seebeck coefficients (figs. S5 and S6), and the n-type conduction would be attributed to the Se vacancy because it is known that Se vacancy contributes to the n-type character of PbSe (*19*). On the other hand, only the pure SnSe film shows highly resistive p-type conduction due to a small amount of intrinsic Sn vacancy (*33*). The carrier transport analysis for pure PbSe and SnSe films is explained in figs. S7 and S8, respectively. The ρ-*T* curves of the (Pb_0.5_Sn_0.5_)Se alloy films exhibit large ρ jumps around ρ transition temperature (*T*_tran._), and similar behaviors are observed in other films with *x* = 0.31 and 0.43. We confirmed that the ρ jumps are observable under the first and the second cooling cycles. Here, we define *T*_tran._ as the temperature with the steepest gradient dρ/d*T* as shown in the inset of Fig. 4 and plot *T*_tran._ as a function of *x* in Fig. 4. *T*_tran._ continuously shifts from 48 to 143 K for the cooling process and from 48 to 255 K for the heating process as *x* increases, showing that *T*_tran._ increases with increasing *x*, accompanying the enhanced hysteresis (*T*_tran.,cooling_ – *T*_tran.,heating_). The large hysteresis would originate from different bond dissociation energy for the threefold coordinated Sn─Se = 401.2 kJ/mol and sixfold coordinated Pb─Se = 302.9 kJ/mol (*34*) during the structure phase transition, as discussed later. This indicates that the structure transition from the RS- to the GeS-type phase is easier than that from the GeS- to the RS-type phase.

Figure 3B shows the temperature dependences of *R*_H_. First, we should notice that *R*_H_(*T*) values do not change monotonically and exhibit minima. For example, for the cooling process of *x* = 0.5, *R*_H_(*T*) takes a minimum at *T* = 118 K although *R*_H_ values keep the negative values and show similar values in the low-*T* (<55 K) and the high-*T* regions (>165 K) far from *T*_tran._. Reflecting this anomalously small *R*_H_ at the minimum, the nominal carrier concentration calculated from *R*_H_, *n* = 1/(*eR*_H_), in Fig. 3C shows a maximum, and the Hall mobility calculated by μ = 1/(*e*ρ*n*) in Fig. 3D shows a minimum. Similar behaviors are often observed in narrow-gap semiconductors and semimetals in which both electrons and holes contribute to the Hall effect. In such case, an electron-hole mixed conduction model

$$R_{H}=\frac{1}{e}\frac{\left(n_{h}\mu_{h}^{2}-n_{e}\mu_{e}^{2}\right)}{{\left(n_{h}\mu_{h}+n_{e}\mu_{e}\right)}^{2}}$$

should be applied, where *n*_e_ and *n*_h_ are electron and hole concentrations, respectively, and μ_e_ and μ_h_ are electron and hole mobility, respectively. In the electron-hole mixed conduction case, *R*_H_ value crosses zero when the Fermi level (*E*_F_) shifts and crosses the condition $n_{h}\mu_{h}^{2}\approx n_{e}\mu_{e}^{2}$, and shows a maximum in *n* = 1/(*eR*_H_). On the other hand, although the sign of *R*_H_ for the electron-hole mixed conduction model should be inverted between both the sides of the *R*_H_ peak because the sign of $n_{h}\mu_{h}^{2}-n_{e}\mu_{e}^{2}$ flips, such *R*_H_ sign inversion is not observed for the present (Pb_0.5_Sn_0.5_)Se case. Further, if we virtually assume a monotonic *R*_H_ variation with *T*, then μ still takes a clear minimum, as shown in fig. S9. These results indicate that the *R*_H_ minima and nonmonotonous variation are not from the mixed conduction, and the μ minimum is not an artifact of the Hall analysis and reflects a real transport mechanism. As will be seen in the next section, the *R*_H_ minimum peak region corresponds to the gradual structural phase transition. It suggests that the *R*_H_ minimum is related to the nonhomogeneous electronic states formed by the microstructure of mixed phases of the gapless RS-type phase and the wider bandgap GeS-type phase around the temperature range. A possible explanation is as follows. A part of the RS-type phase with the gapless Dirac-like state gradually transformed into a wider-bandgap GeS-type phase, and the conduction band of the RS-type phase will be confined by the GeS-type phase, which confines the mobile carriers in the RS-type phase, decreasing μ down to ~110 K. Further decreasing the *T* increases the volume fraction of the GeS-type phase and lastly gives rise to μ ~ 20 cm^2^/(V·s) of the GeS-type phase. When GeS-type phase becomes dominant at low *T*, the μ increases with a decrease of *T*, indicating that grain boundary scattering does not affect the carrier transport (note that grain boundary potential results in thermally activated behaviors in mobility).

Because of the complex situation in the transition state, we will not get into a deeper discussion in this issue hereafter. On the other hand, we should notice that the Hall effect results are reliable at both the high-temperature limit and the low-temperature limit, respectively, because those *E*_F_ are almost temperature independent at ~130 meV and in the single-carrier n-type regime, as will be confirmed by the first-principles calculations later on for section S4.

Hereafter, we focus on the (Pb_0.5_Sn_0.5_)Se (*x* = 0.5) sample that exhibits the largest ρ jump. The ρ-*T* curve in the cooling process (the blue closed circles in Fig. 3A) exhibits a specific variation where ρ exhibits a broad peak between the low-ρ state in the low-*T* region and the high-ρ state in the high-*T* region, where the ρ value changes by three orders of magnitude from the maximum value of 0.4 ohms·cm at 120 K to 10^−3^ ohms·cm at >180 K.

## DISCUSSION

### 3D-2D structural phase transition of (Pb_1−*x*_Sn*_x_*)Se alloy

**Fig. 5:**
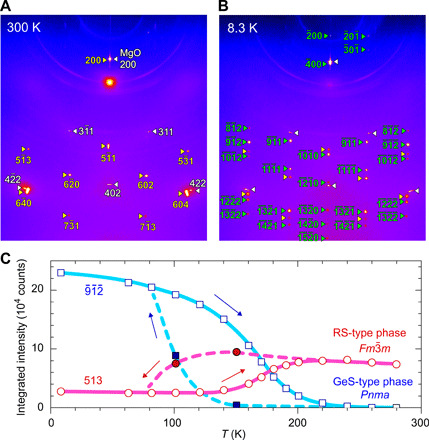
Structure transition of (Pb_1−*x*_Sn*_x_*)Se alloy films. (**A** and **B**) Synchrotron single-crystal XRD patterns measured at (A) *T* = 300 K and (B) 8.3 K for (Pb_0.5_Sn_0.5_)Se epitaxial film. The white left triangles, the yellow right triangles, and the green right triangles indicate the diffraction spots from the MgO substrate, the RS-type phase, and the GeS-type phase, respectively. The diffraction indices for the cubic RS-type phase and the orthorhombic GeS-type phase are indicated in (A) and (B), respectively. (**C**) Temperature dependences of the integrated intensities for the RS-type 513 diffraction in (A) and the GeS-type $\bar{9}\;\bar{1}\;\bar{2}$ diffraction in (B), which were first measured during heating and then measured during cooling process.

To investigate the origin of the giant electronic modulation, we investigated the temperature variation of the crystal structure by single-crystal synchrotron XRD analysis (see fig. S10 for the measurement details) (*35*). Figure 5 (A and B) shows the XRD patterns taken at *T* = 300 K (A) and 8.3 K (B) for the (Pb_0.5_Sn_0.5_)Se film. All the diffraction spots at *T* = 300 K are assigned to the RS-type phase with the space group *Fm*$\bar{3}$*m* (the yellow triangles) and the MgO substrate (the white triangles), which is consistent with the XRD pattern in Fig. 2A. On the other hand, additional diffractions appear at *T* = 8.3 K (the green triangles), which are assigned to the 2D GeS-type phase (*Pnma*), along with the remaining diffraction spots from the 3D RS-type phase (the yellow triangles). The appearance of the additional diffractions was also confirmed in the electron diffraction by TEM at *T* = 100 K (fig. S11). The lattice parameters and the volume of the GeS-type phase were estimated to be *a* = 11.587 Å, *b* = 4.308 Å, *c* = 4.306 Å, and *V* = 214.942 Å^3^ at *T* = 8.3 K. Compared to the lattice parameters of the pure SnSe epitaxial film on MgO (*a* = 11.54 Å, *b* = 4.19 Å, *c* = 4.37 Å, and *V* = 221.30 Å^3^), the GeS-type (Pb_0.5_Sn_0.5_)Se film has higher tetragonality with almost the same values in the *b*- and *c*-axis lattice parameters. The lattice parameter of the $\sqrt{2}\times \sqrt{2}$ supercell unit, i.e., $\sqrt{b^{2}+c^{2}}$ = 6.091 Å is larger than 6.043 Å of the RS-type phase at *T* = 8.3 K.

We confirmed that this structural change is reversible and repeatable. To semiquantitatively show the variation of the amount of each phase, the integrated intensities of the GeS-type phase $\bar{9}\;\bar{1}\;\bar{2}$ diffraction $I_{\bar{9}\;\bar{1}\;\bar{2},\text{GeS}}$ and the RS-type phase 513 diffraction *I*_513, RS_ are plotted against *T* in Fig. 5C. Note that their crystal structure factors are $|F_{\bar{9}\bar{1}\bar{2},\text{GeS}}|=121$ and ∣*F*_513, RS_∣ = 180, respectively, and the phase-fraction ratio is roughly estimated by $[\text{RS}]:[\text{GeS}]=I_{513,\text{RS}}/{|F_{\bar{9}\;\bar{1}\;\bar{2},\text{GeS}}|}^{2}:I_{\bar{9}\;\bar{1}\;\bar{2},\text{GeS}}/{|F_{513,\text{RS}}|}^{2}$. It indicates that the phase fraction of the RS-type phase is ~100% at RT, and ~80% of the RS-type phase is transformed to the GeS-type phase at *T* = 8.3 K. For the cooling process, the RS- to the GeS-type phase transition starts from *T* = ~150 K and completes down to *T* = 80 K. This temperature range corresponds to the large ρ jump and its maximum in Fig. 3A, implying that the giant modulation in the electronic properties is ascribed to the structural phase transition and the associated electronic structure transition. Note that we compared the 400(C) out-of-plane XRD peak profiles of the Pb_0.5_Sn_0.5_Se epitaxial film before and after the cooling-heating cycles (fig. S12), showing that the diffraction angle and the peak width were not changed. This result indicates that the film structure including crystallite size and strain were not changed before and after the cooling-heating cycles, supporting that spinodal decomposition does not occur in Pb_0.5_Sn_0.5_Se epitaxial film by heating up to 335 K.

### Electronic structure of (Pb_1−*x*_Sn*_x_*)Se alloy

To investigate the electronic structure change and its effect on the giant electronic modulation, we performed first-principles hybrid density functional theory (DFT) calculations, where ground-state stable structures and electronic structures are calculated using the Vienna Ab Initio Simulation Package (VASP) code with spin-orbit interaction and carrier transport properties (Hall coefficient and conductivity/relaxation time ratio σ/τ_0_) calculated using the BoltzTraP2 code based on full-band transport equation (*36*). For structural relaxation, atomic coordinates were relaxed with the lattice parameters fixed to the experimental values for the RS-type phase and the GeS-type phase at *T* = 8.3 K. The relaxed crystal structure models are given in fig. S13. In preliminary calculations, we examined the generalized gradient approximation–Perdew-Burke-Ernzerhof (GGA-PBE) and hybrid Heyd-Scuseria-Ernzerhof (HSE06) functionals but found that only HSE06 can explain the observed *R*_H_ value at RT for the RS-type (Pb_0.5_Sn_0.5_)Se (*R*_H_ = −0.67 cm^3^/C); for example, the GGA-PBE shows a large overlap of the conduction band and valence band with a negative bandgap, and therefore, the minimum density of states (DOS) is somewhat high and the |*R*_H_| maximum does not exceed 0.16 cm^3^/C. Using the HSE06 hybrid functional, the conduction band is slightly upshift and the valence band vice versa, reducing the minimum DOS value (fig. S14A) and leading to a larger |*R*_H_| maximum of 18 cm^3^/C (fig. S14B), being consistent with the observed *R*_H_.

**Fig. 6:**
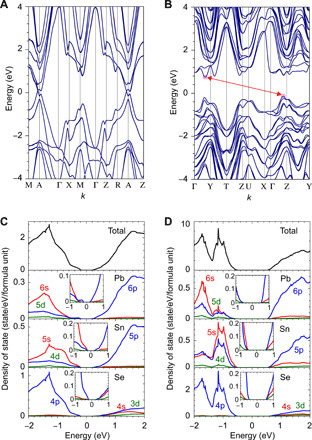
Electronic structure of (Pb_1−*x*_Sn*_x_*)Se alloy. (**A** to **D**) Electronic structures of (Pb_0.5_Sn_0.5_)Se for (A and C) RS-type phase and (B and D) GeS-type phase calculated using the HSE06 hybrid functional with spin-orbit interaction. Energy is measured from the ground-state Fermi level. (A and B) Band structures and (C and D) the DOSs and partial DOSs projected on each element and orbitals of Pb, Sn, and Se.

Figure 6 (A and B) shows the band structures of (Pb_0.5_Sn_0.5_)Se for the RS-type phase (A) and the GeS-type phase (B). The DOSs and partial DOSs projected on each element and orbitals of Pb, Sn, and Se are shown in Fig. 6 (C and D). It indicates that the RS-type phase (Pb_1−*x*_Sn*_x_*)Se film has a gapless Dirac-like state around the energy level ~100 meV, where the upper and the lower bands are split by the spin-orbit interaction and exhibits two sharp minima and maxima, respectively. Upon the phase transition from the RS-type to the GeS-type phase, the Pb 6s and Sn 5s orbitals shift down while Pb 6p and Sn 5p orbitals shift up, which opens the indirect bandgap with *E*_g_ = 0.28 eV in the GeS-type phase.

The electron effective mass for the GeS-type phase is estimated to be *m*_e_* = 0.12 (at the A point) to 0.23 *m*_0_ (in the A to Γ line) from the conduction band edge band. On the other hand, estimation of *m*_e_* for the RS-type phase is complicated because multiple electron/hole bands exist near 0 eV and would depend on *E*_F_ (note that this *E*_F_ is the electron chemical potential in the material and not the DFT ground state *E*_F_ = 0 eV). As explained in section S4, *E*_F_ = 130 meV is plausible and provides *m*_e_* values ranging from 0.01 to 0.13 *m*_0_. This result substantiates that *m*_e_* are very different by a factor of ~10 for the RS-type phase and the GeS-type phase and supports the conclusion that the observed giant electronic property modulation at *T*_trans._ originates from the large mobility change due to the band structure modification while the contribution of carrier concentration is as small as a factor of 3 (*n* are ~3 × 10^18^ and 1 × 10^19^ cm^−3^ in the low-*T* and high-*T* regions in Fig. 3C, respectively).

In summary, we artificially induce the 3D-2D structure phase transition and demonstrate giant electronic property modulation in nonequilibrium alloy (Pb_1−*x*_Sn*_x_*)Se. The MPB between cubic 3D PbSe phase and layered 2D SnSe phase is produced in the (Pb_1−*x*_Sn*_x_*)Se alloy, which is realized by the nonequilibrium two-step process that combined the R-SPE with rapid quenching. It is found that the (Pb_1−*x*_Sn*_x_*)Se induces the direct phase transition from 3D to 2D structure at around the critical composition *x* = 0.5. The 3D-to-2D structure transition is reversibly controlled by temperature, and more than three orders of magnitude change of electron mobility is observed because of the distinct band structure change from Dirac state to semiconducting state. The present strategy, facilitating different structure dimensionality switching, would realize further functional phase switching using artificial MPB. For example, the exotic surface state of the topological crystalline insulator of (Pb_1−*x*_Sn*_x_*)Se serves as a platform for fundamental scientific studies and future electronics such as low-dissipation electronics and spintronics (*14*, *15*, *37*–*40*). The topological surface state is protected by the crystalline symmetry in the cubic RS-type phase, and thus, the 3D-2D structure transition would give a novel function of topological state switching.

## MATERIALS AND METHODS

### Nonequilibrium film growth

The (Pb_1−*x*_Sn*_x_*)Se epitaxial films were fabricated by the two-step process combining R-SPE and subsequent thermal quenching. First, a SnSe/PbSe bilayer film structure was formed on a MgO (100) single-crystal substrate by pulsed laser deposition (PLD) in a vacuum. The PbSe epitaxial film was deposited on MgO at 500°C to serve as an epitaxial template layer, and then the SnSe layer was sequentially deposited on PbSe/MgO at RT. The total film thickness of the bilayer structure was controlled to ~50 nm. A KrF excimer laser (wavelength, 248 nm; repetition rate, 10 Hz) was used to ablate Se-rich PbSe_1.2_ and SnSe_1.2_ polycrystalline target disks, where the chemical compositions were optimized so as to obtain the best epitaxial films. The base pressure of the PLD growth chamber was ~1 × 10^−5^ Pa. Laser fluence for ablation was fixed at 1.5 J/cm^2^. The resulting bilayer film was covered with a fresh MgO single-crystal plate, which is effective for preventing evaporation of the film constituents during thermal annealing. The covered film structure was sealed in an Ar-filled silica-glass ampule (~1 atm) and then annealed at 600°C for 30 min. After that, the ampule was subjected to rapid quenching in iced water to freeze the high-temperature RS-type (Pb_1−*x*_Sn*_x_*)Se phase. In this method, the formation of the PbSe epitaxial template layer is a key to achieving the epitaxial growth, and the annealing process converted the SnSe/PbSe bilayer to a uniform epitaxial film.

### Structural and electronic property characterization

Crystal structures and orientations were investigated by high-resolution XRD with monochromatic CuKα_1_ anode radiation (SmartLab, Rigaku Co.). Chemical compositions of the films (i.e., atomic ratios of Sn, Pb, and Se) were evaluated with a field-emission electron probe microanalysis with the beam radius of 5 nm and the spatial resolution of ~20 nm for 50-nm-thick films confirmed by simulation. A cross-sectional atomic-resolution image and an electron diffraction pattern of the film were examined by high-resolution STEM and TEM (JEM-ARM200F, JEOL Ltd.), where the electron beam was incident parallel to MgO [001]. The cross-sectional chemical composition was observed by STEM-EDS with the spatial resolution of ~1 nm. The sample was prepared by mechanical polishing and Ar^+^ ion milling. Synchrotron XRD experiments were performed with the x-ray wavelength of 0.685374 Å at BL-8B beamline at the Photon Factory of the High Energy Accelerator Research Organization. Intensity data were collected by an imaging plate as an area detector (Rigaku R-AXIS). We initially checked the structural phase transition by reducing the *T* from 300 to 8.3 K. After that, we took x-ray pictures at *T* from 8.3 to 300 K during heating and then at *T* = 150 and 100 K during the cooling process. We confirmed that the phase transition is reversible by repeating the cooling and heating process. Electronic properties were measured by the Hall effect using the van der Pauw configuration, where the Pt electrode was used for ohmic contact, where we measured current-voltage (*I*-*V*) characteristics and confirmed their linearity at all temperatures in ρ-*T* curves.

### Density functional theory calculation

The band structures and DOSs of (Pb_0.5_Sn_0.5_)Se were calculated on the basis of DFT, conducted using the projection-augmented wave (PAW) method as implemented in the VASP. For the exchange-correlational potential, we used the HSE06 hybrid functional. The adopted PAW potentials treated Sn [4d5s5p], Pb [5d6s6p], and Se [4s4p] orbitals as valence states. A plane wave cutoff energy of 500 eV and a Γ-centered *k*-mesh (4 × 10 × 10 *k*-mesh for the GeS-type phase and 6 × 10 × 10 *k*-mesh for a primitive cell of the RS-type phase) were used for the calculations of the ground-state crystal structure. The lattice parameters were constrained to the experimental values (*a* = 11.587 Å, *b* = 4.308 Å, and *c* = 4.306 Å for the GeS-type phase and *a* = 6.043 Å for the RS-type phase). The internal atomic coordinates were fully relaxed until all the forces on the atoms became less than 0.01 eV/Å and the total energy difference was smaller than 10^−6^ eV. The spin-orbit coupling was taken into account in all of the calculations. The electronic transport properties were calculated on the basis of the Bloch-Boltzmann theory, as implemented in the BoltzTraP2 code (*36*). For drawing the band structures with the HSE06 functional, we used the BoltzTraP2 code for the GeS-type phase. For the RS-type phase, we performed the Wannier interpolation by using the Wannier90 code (*41*), with which we were able to correctly describe the band inversion near the Fermi energy. The first Brillouin zone internal coordinates are Γ(0 0 0), Y(0 0.5 0), T(0 0.5 0.5), Z(0 0 0.5), U(0.5 0 0.5), and X(0.5 0 0) for the GeS-type phase and M(0.5 0.25 −0.25), A(0.5 0.5 0), Γ(0 0 0), X(0 0.25 −0.25), Z(0 0.25 0.25), and R(0 0.5 0) for the RS-type phase.

## Supplementary Material

http://advances.sciencemag.org/cgi/content/full/7/12/eabf2725/DC1

Adobe PDF - abf2725_SM.pdf

Reversible 3D-2D structural phase transition and giant electronic modulation in nonequilibrium alloy semiconductor, lead-tin-selenide

## SUPPLEMENTARY MATERIALS

Supplementary material for this article is available at http://advances.sciencemag.org/cgi/content/full/7/12/eabf2725/DC1
