# Supplementary material for: Reversible 3D-2D structural phase transition and giant electronic modulation in nonequilibrium alloy semiconductor, lead-tin-selenide
Source: Sci Adv. 2021 Mar 19;7(12):eabf2725. doi: 10.1126/sciadv.abf2725 (PMC7978423; doi:10.1126/sciadv.abf2725)
Supplement: http://advances.sciencemag.org/cgi/content/full/7/12/eabf2725/DC1 [file supp_7_12_eabf2725__7.12.eabf2725.DC1.html]

Science Advances | Science AdvancesAAASSearchScience AdvancesMenu

## Supplementary Materials

# Reversible 3D-2D structural phase transition and giant electronic modulation in nonequilibrium alloy semiconductor, lead-tin-selenide

Takayoshi Katase, Yudai Takahashi, Xinyi He, Terumasa Tadano, Keisuke Ide, Hideto Yoshida, Shiro Kawachi, Jun-ichi Yamaura, Masato Sasase, Hidenori Hiramatsu, Hideo Hosono, Toshio Kamiya

Download Supplement

**This PDF file includes:**

- Sections S1 to S4
- Figs. S1 to S15
- References

**Files in this Data Supplement:**

- Adobe PDF - abf2725\_SM.pdf
