# Supplementary material for: Reversible 3D-2D structural phase transition and giant electronic modulation in nonequilibrium alloy semiconductor, lead-tin-selenide
Source: Sci Adv. 2021 Mar 19;7(12):eabf2725. doi: 10.1126/sciadv.abf2725 (PMC7978423; doi:10.1126/sciadv.abf2725)
Supplement: http://advances.sciencemag.org/cgi/content/full/7/12/eabf2725/DC1 [file supp_7_12_eabf2725__abf2725_SM.pdf]

Science Advances
AAAS

advances.sciencemag.org/cgi/content/full/7/12/eabf2725/DC1

Supplementary Materials for

Reversible 3D-2D structural phase transition and giant electronic modulation in nonequilibrium alloy semiconductor, lead-tin-selenide

Takayoshi Katase*, Yudai Takahashi, Xinyi He, Terumasa Tadano, Keisuke Ide, Hideto Yoshida, Shiro Kawachi, Jun-ichi Yamaura, Masato Sasase, Hidenori Hiramatsu, Hideo Hosono, Toshio Kamiya*

*Corresponding author. Email: katase@mces.titech.ac.jp (T.Kat.); kamiya.t.aa@m.titech.ac.jp (T.Kam.)

Published 19 March 2021, *Sci. Adv.* **7**, eabf2725 (2021)
DOI: 10.1126/sciadv.abf2725

This PDF file includes:

Sections S1 to S4
Figs. S1 to S15
References

Section S1. Epitaxial growth and structure characterization of ($Pb_{1-x}Sn_x$)Se alloy films

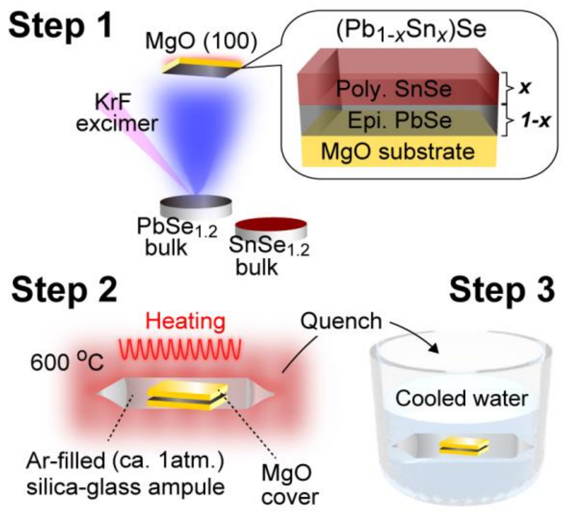

Fig. S1. Schematic procedure of the two-step process combining R-SPE method with rapid quenching. SnSe/PbSe bilayer film structure was deposited by PLD (Step 1). At first, the epitaxial template layer of PbSe was deposited at a growth temperature of 500 °C, and then the SnSe layer was sequentially deposited at RT. The surface of the bilayer film was covered with a fresh MgO plate to prevent the evaporation of constituent elements during thermal annealing (Step 2). Then, the bilayered film was sealed in an Ar-filled silica-glass ampule (~1 atm). The sealed ampule was annealed at 600 °C for 30 min. Then the ampule was rapidly quenched in cooled water from 600 °C to RT in order to stabilize the high-temperature RS-type ($Pb_{1-x}Sn_x$)Se film (Step 3).

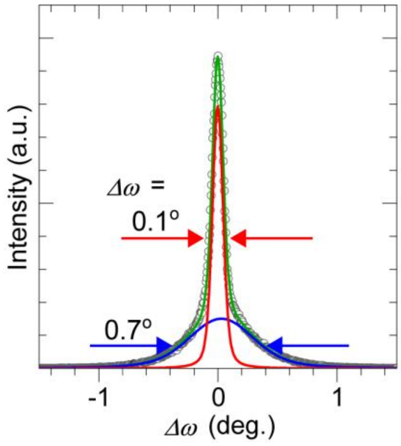

Fig. S2. Out-of-plane rocking curve of the cubic 200 diffraction of ($Pb_{0.5}Sn_{0.5}$)Se epitaxial film. The circles and the lines show the observed pattern and peak deconvolution results, respectively. The green line indicates the total fitting curve. The rocking curve is composed of sharp and broad peaks with the different full width at half maximum (FWHM) values of 0.1°(red line) and 0.7° (blue line), respectively.

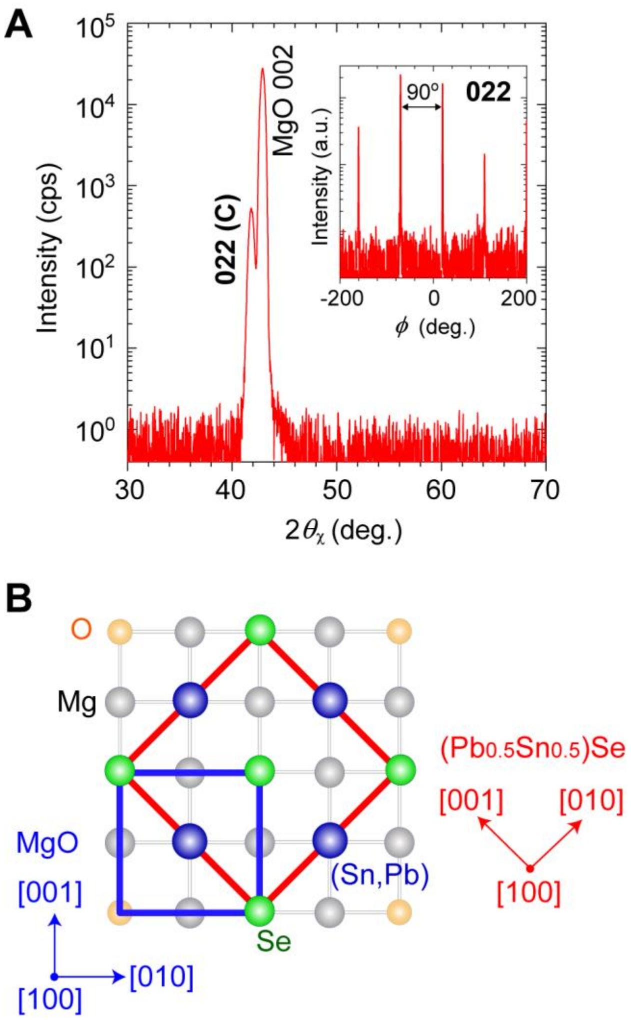

Fig. S3. (**A**) In-plane XRD pattern of $(Pb_{0.5}Sn_{0.5})Se$ film on MgO (100) substrate. Inset shows the in-plane ϕ scan of the 022 diffraction of the $(Pb_{0.5}Sn_{0.5})Se$ film. The $(Pb_{0.5}Sn_{0.5})Se$ 022 and MgO 002 diffraction peaks are observed, and the in-plane ϕ scan of $(Pb_{0.5}Sn_{0.5})Se$ 022 diffraction shows a single-domain 4-fold rotational symmetry due to the cubic lattice, which confirms the epitaxial relationship of $(Pb_{0.5}Sn_{0.5})Se$ [100] || MgO [100] for the out-of-plane and $(Pb_{0.5}Sn_{0.5})Se$ [011] || MgO [001] for the in-plane. (**B**) Schematic in-plane epitaxial relationship between the film and MgO (100) substrate. The $(Pb_{0.5}Sn_{0.5})Se$ and MgO unit cells are drawn by the red and the blue squares, respectively.

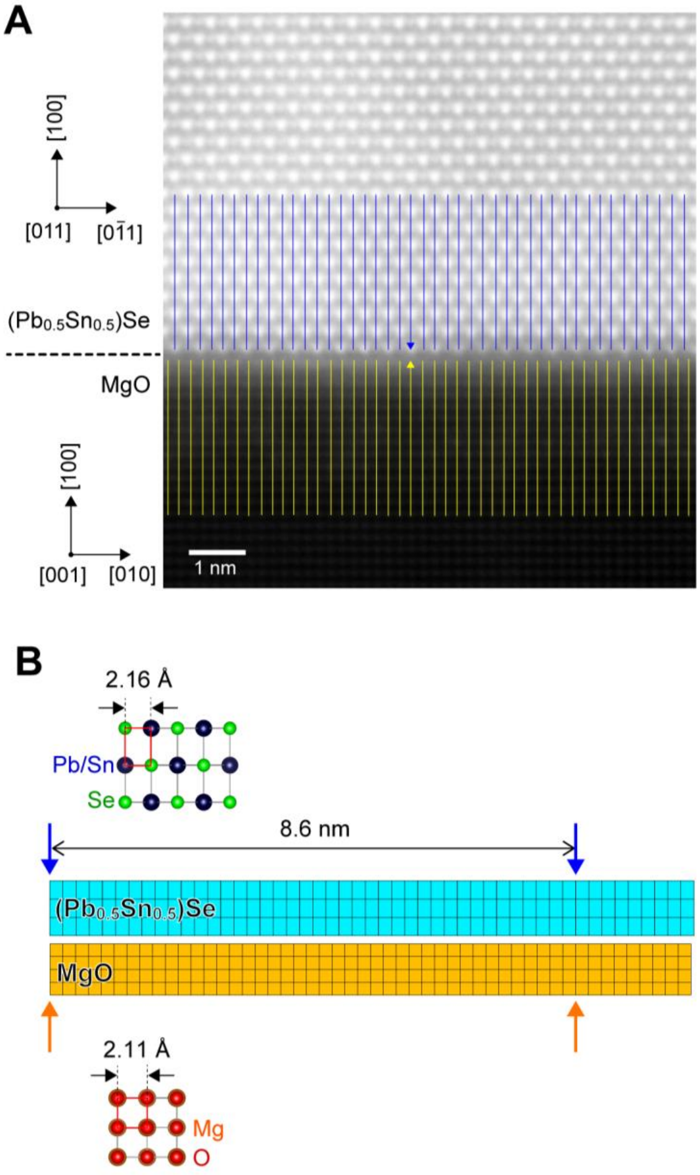

Fig. S4. (**A**) Cross-sectional HAADF-STEM image for $(Pb_{0.5}Sn_{0.5})Se$ film on MgO (100) substrate. The horizontal dotted line indicates the film/substrate interface position. The clear atomic structure of RS-type phase is seen from the vicinity of the interface to bulk region, where the pseudo-semi coherent interface is formed at the hetero-interface of $(Pb_{0.5}Sn_{0.5})Se$ on MgO, as indicated by arrows. (**B**) Interface structure model for $(Pb_{0.5}Sn_{0.5})Se$ and MgO, where the coherent atomic column position with an interval of 8.6 nm is marked by arrows.

Section S2. Electrical properties and analysis of carrier transport mechanism for $(Pb_{1-x}Sn_x)Se$ films

Figure S5 summarizes the complete set of the electrical properties of $(Pb_{1-x}Sn_x)Se$ films (i.e., it adds x = 0.31 and 0.43 to **Figs. 3(A-D)**) and their variations with x. **Figure S6** shows Seebeck coefficient at RT. As explained for x = 0.5, the x = 0.31 – 0.5 films exhibit ρ jumps, R_H maxima, and μ minima. The right figures show that ρ at RT gradually decreases with increasing x, where μ is increased presumably due to the band gap narrowing and the associated enhancement in the conduction band dispersion (*16*).

For the pure cubic PbSe (x = 0) film, ρ monotonously increases with increasing temperature, which originates from the rather large decrease in μ irrespective of the slight increase in n, where the μ follows $T^{-3/2}$ dependence at T > 200 K (**Fig. S7**) due to the acoustic-phonon scattering and similar μ decrease is observed in PbSe single crystals as reported in ref. *42*. On the other hand, the orthorhombic SnSe (x = 1.0) film shows a large decrease in ρ with increasing T, where both n and μ increase. The Arrhenius plot of n for the pure SnSe film is shown in **Fig. S8**, where the activation energy (E_a) is estimated to be 198 meV, corresponding to the acceptor level from the valence band maximum of $E_d = 2E_a$ = 396 meV.

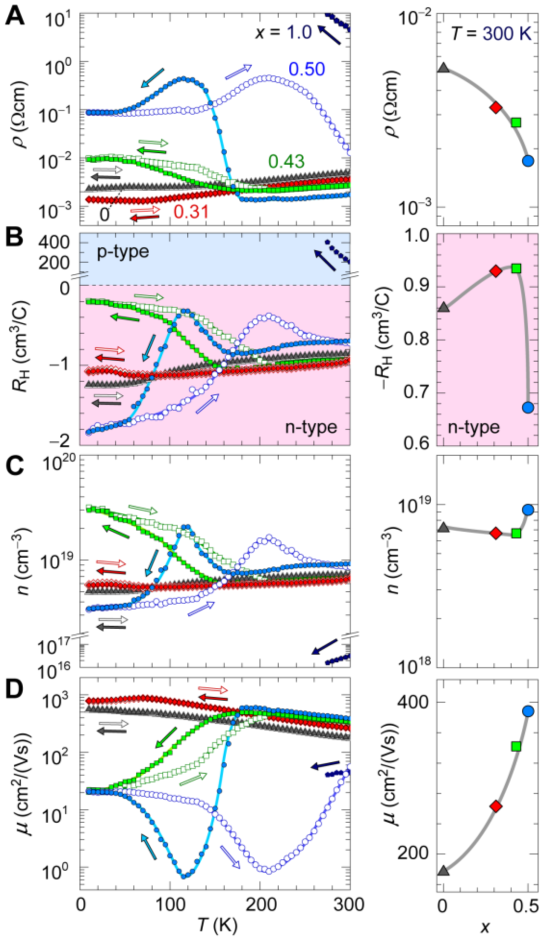

Fig. S5. (**A-D**) Temperature dependences of (**A**) resistivity (ρ), (**B**) Hall coefficient (R_H), (**C**)

nominal carrier concentration ($n = 1 / (eR_H)$), and (**D**) Hall mobility ($\mu = 1 / (en\rho)$) for ($Pb_{1-x}Sn_x$)Se epitaxial films. Each measurement was started from the cooling process (the closed symbols) and then switched to the heating process (the open symbols). The right panels show the x dependence of ρ, R_H, n, and μ measured at RT before starting the cooling measurements. Red and blue areas in (**B**) indicate n-type and p-type conduction, respectively.

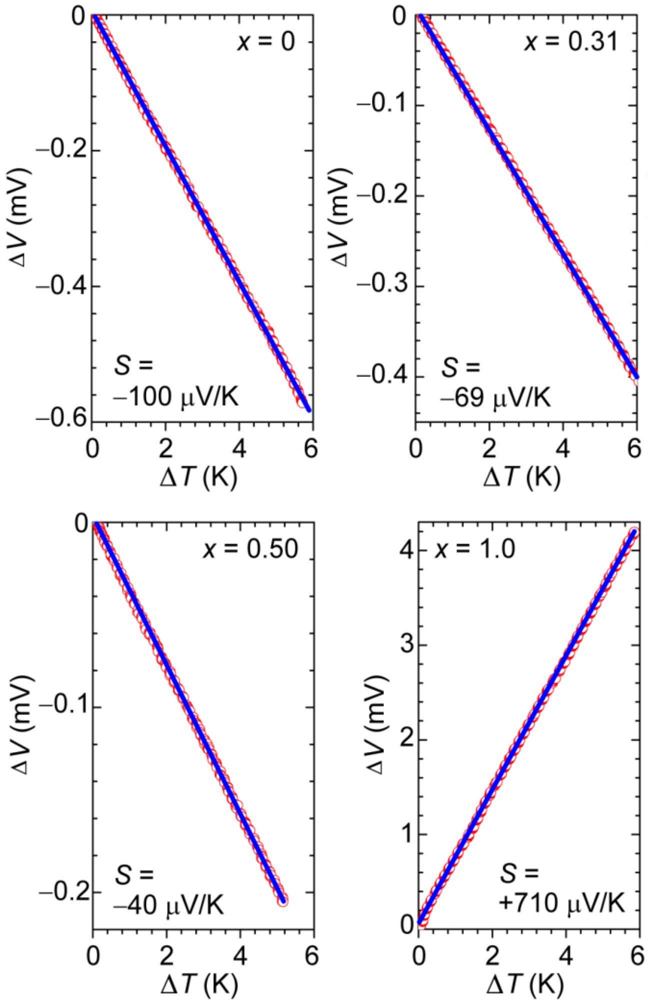

Fig. S6. Temperature difference (ΔT) vs. thermo-electromotive force (ΔV) for ($Pb_{1-x}Sn_x$)Se epitaxial films at room temperature. The ΔV and ΔT were simultaneously measured by varying ΔT up to ~6 K along the in-plane direction, and the Seebeck coefficient (S) was obtained from the linear slope (blue line) of the ΔV–ΔT plots (red open circles).

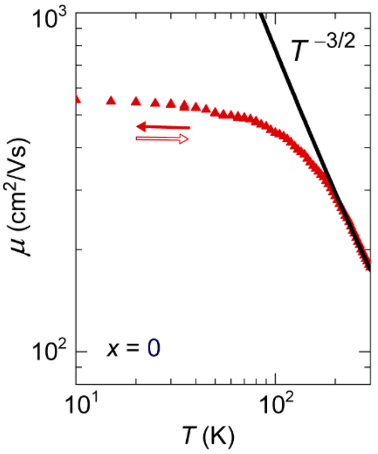

Fig. S7. Temperature (T) dependence of mobility (μ) for pure PnSe (x = 0) epitaxial film. The μ follows $T^{-3/2}$ dependence at T > 200 K due to the acoustic-phonon scattering.

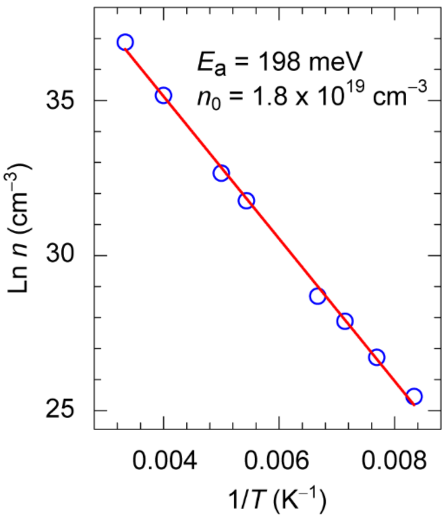

Fig. S8. Arrhenius plot of Ln n vs. 1/T for pure SnSe epitaxial film. The activation energy (E_a) and intrinsic hole concentration (n_0) calculated from the slope using the relation of $\mathrm{Ln}\, n = -\frac{E_a}{k_B T} + \mathrm{Ln}\, n_0$, where k_B is Boltzmann's constant, are described in the inset.

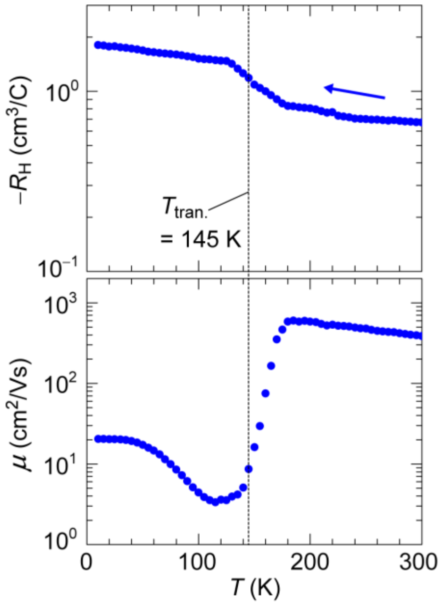

Fig. S9. (Top figure) Virtually assumed monotonic $R_H(T)$ modified from the data in **Fig. 3(b)** for the cooling process (the closed blue circles). Even if the monotonic variation is assumed for $R_H(T)$, μ still exhibits a clear minimum as seen in the bottom figure.

Section S3. Temperature dependence of crystal structure for $(Pb_{1-x}Sn_x)Se$ alloy films

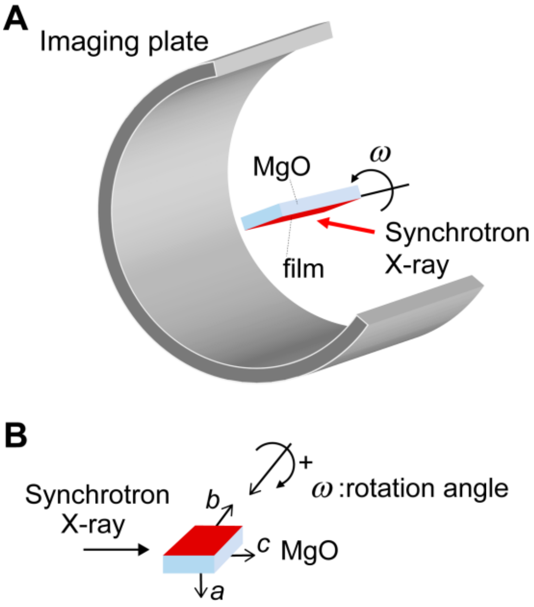

Fig. S10. (**A**) Schematic setup of synchrotron single-crystal XRD analysis, where the x-ray photographs were taken on a curved imaging plate with the sample oscillation in the ω range of 0.6 – 8° during the synchrotron x-ray exposure. (**B**) Geometrical relationship of x-ray incident angle and the crystal orientation of the MgO substrate.

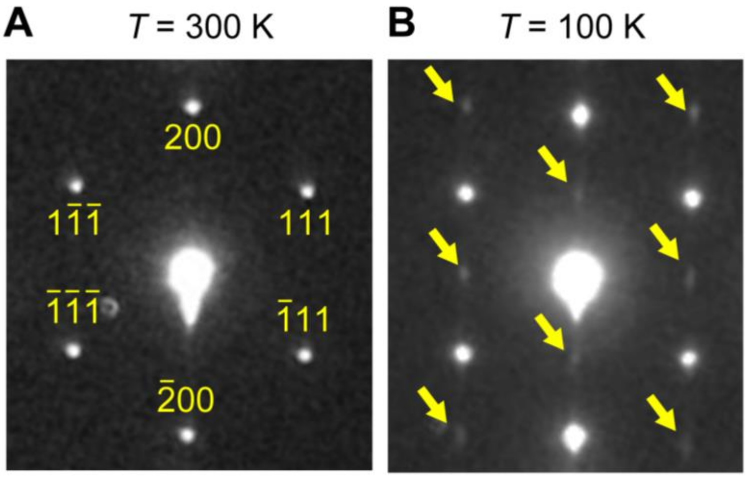

Fig. S11. Electron diffraction patterns taken at T = 300 K (**A**) and 100 K (**b**) for $(Pb_{0.5}Sn_{0.5})Se$ epitaxial film by TEM. The main diffraction indices of the RS-type phase are indicated in (**A**). The arrows in (**B**) indicate the superlattice diffraction spots, which appear due to structural transformation from the RS-type to the GeS-type structure.

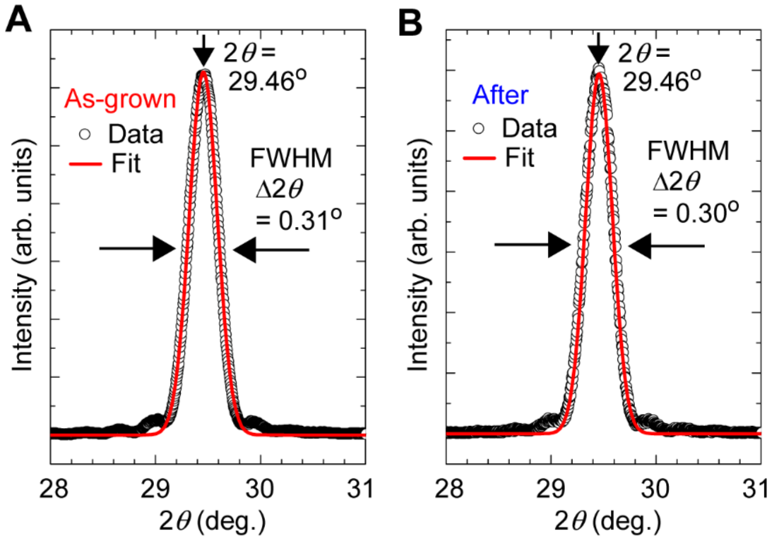

Fig. S12. (A,B) Out-of-plane XRD patterns around 400(C) diffraction measured before (A) and after 3-times cooling-heating cycles (B) for $Pb_{0.5}Sn_{0.5}Se$ epitaxial film.

Section S4. Density functional theory calculations

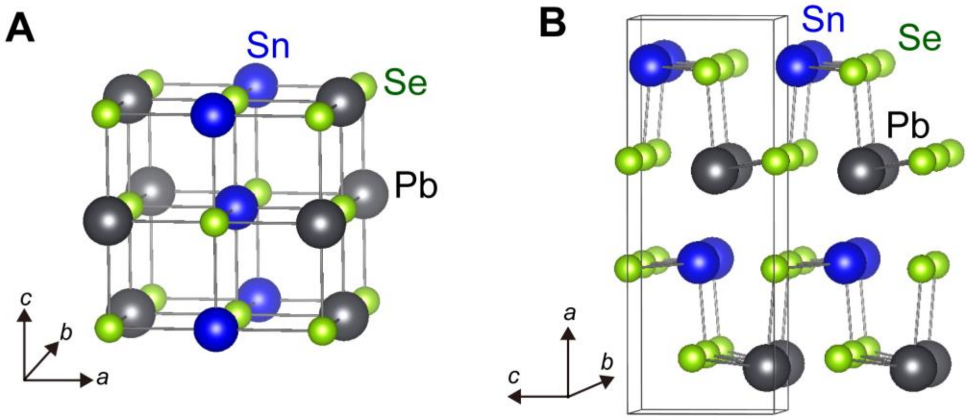

Fig. S13. Relaxed structures of $(Pb_{0.5}Sn_{0.5})Se$ with RS-type (**A**) and GeS-type structure (**B**), calculated using GGA-PBE functional.

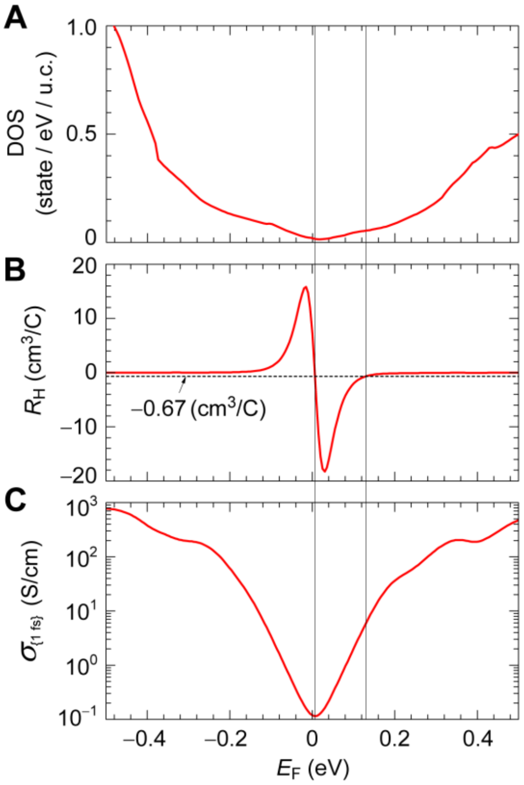

Fig. S14. Calculated (**A**) density of states, (**B**) Hall coefficient (R_H), and conductivity for the relaxation time 1 fs ($\sigma_{\{1\ fs\}}$) as a function of electron chemical potential E_F using HSE06 hybrid functional with spin-orbit interaction for RS-type $(Pb_{0.5}Sn_{0.5})Se$. E_F is measured from the Fermi energy given by the HSE06 ground state. The horizontal dotted line in (**B**) shows the experimental R_H value at RT, and the vertical lines show the corresponding E_F.

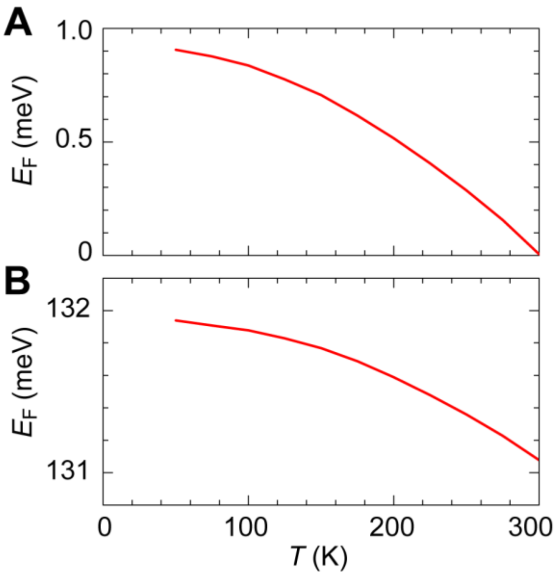

Fig. S15. Temperature dependences of E_F for RS-type phase $(Pb_{0.5}Sn_{0.5})Se$ calculated using the DOS in **Fig. 5(C)** and semiconductor statistics with the condition to keep the total electron number. E_F are chosen to reproduce the experimental R_H value at RT in **Fig. S14(B)**, E_F(RT) = (**A**) 6 meV and (**B**) 130 meV.

Figure S14(B) gives two possible E_F(RT), 6 meV or 130 meV, from the measured RT R_H value of –0.67 cm^3/C (indicated by the cross-sections with the horizontal dotted line corresponding to the RT R_H value). We also confirmed that the variation of E_F with temperature in the range 50 – 300 K is smaller than 7 meV and negligible for the following discussion (**Fig. S15**). Then, m_e^* are estimated from the bands near 6 – 130 meV. The RS-type phase band structure in **Fig. 5(A)** has four minima around the A point at ~ 101 (in M – A), 81 (in A – Γ), 72 (in R – A), 50 meV (in A – Z) with m_e^* = 0.014m_0, 0.051m_0, 0.010m_0, 0.046m_0, respectively. If we employ the m_e^* values at E_F = 130 meV, ~0.13m_0 is obtained. By using the RT Hall electron mobility of μ = 400 cm^2/(Vs) in **Fig. 3(D)** and the representative m_e^* values of 0.01 – 0.13m_0, the momentum relaxation time τ_0 is estimated to be $\tau_0 = m_e{}^*\mu/e$ = 2 – 30 fs. Applying this value to **Fig. S14(C)**, two possible conductivities are obtained to be σ(RT) = 0.3 – 4.0 S/cm and 14 – 200 S/cm for E_F = 6 meV and 130 meV, respectively. Then, we conclude that E_F = 130 meV and m_e^* = 0.13m_0 is more plausible by comparing with the measured σ(RT) of 500 S/cm from **Fig. 3(A)**.

REFERENCES AND NOTES

1. J. T. S. Irvine, M. G. Johnston, W. T. A. Harrison, Lone-pair containment in closed cavities. The MTe_6O_{13} (M = Mn, Ni, Co) family of ternary oxides. *Dalton Trans.*, 2641–2645 (2003).

2. S. V. Krivovichev, T. Armbruster, W. Depmeier, One-dimensional lone electron pair micelles in the crystal structure of $Pb_5(SiO_4)(VO_4)_2$. *Mater. Res. Bull.* **39**, 1717–1722 (2004).

3. S. Sallis, L. F. Piper, J. Francis, J. Tate, H. Hiramatsu, T. Kamiya, H. Hosono, Role of lone pair electrons in determining the optoelectronic properties of BiCuOSe. *Phys. Rev. B* **85**, 085207 (2012).

4. Z. Tian, C. Guo, M. Zhao, R. Li, J. Xue, Two-dimensional SnS: A phosphorene analogue with strong in-plane electronic anisotropy. *ACS Nano* **11**, 2219–2226 (2017).

5. R. Sen, P. Johari, One-dimensional-Sn_2X_3 (X = S, Se) as promising optoelectronic and thermoelectonic materials: A comparison with three-dimensional-Sn_2X_3. *ACS Appl. Mater. Interfaces* **11**, 12733–12744 (2019).

6. J. C. Jamieson, Crystal structures adopted by black phosphorus at high pressures. *Science* **139**, 1291–1292 (1963).

7. S. Wang, C. Zhang, Y. Wang, L. Wang, J. Zhang, C. Childs, H. Ge, H. Xu, H. Chen, D. He, Y. Zhao, Revisit of pressure-induced phase transition in PbSe: Crystal structure, and thermoelastic and electrical properties. *Inorg. Chem.* **54**, 4981–4989 (2015).

8. A. Walsh, G. W. Watson, The origin of the stereochemically active Pb(II) lone pair: DFT calculations on PbO and PbS. *J. Solid State Chem.* **178**, 1422–1428 (2005).

9. L. S. Ramsdell, The crystal structure of some metallic sulfides. *Am. Mineral.* **10**, 281–304 (1925).

10. H. Overhof, U. Rössler, Electronic structure of PbS, PbSe, and PbTe. *Phys. Status Solidi B* **37**, 691–698 (1970).

11. A. Aziza, E. Amzallag, M. Balkanski, Free electron effective mass in PbSe and Pb□SnSe mixed crystals. *Solid State Commun.* **8**, 873–877 (1970).

12. W. H. Strehlow, E. L. Cook, Compilation of energy band gaps in elemental and binary compound semiconductors and insulators. *J. Phys. Chem. Ref. Data Monogr.* **2**, 163–200 (1973).

13. N. Suzuki, K. Sawai, S. Adachi, Optical properties of PbSe. *J. Appl. Phys.* **77**, 1249–1255 (1995).

14. P. Dziawa, B. J. Kowalski, K. Dybko, R. Buczko, A. Szczerbakow, M. Szot, E. Łusakowska, T. Balasubramanian, B. M. Wojek, M. H. Berntsen, O. Tjernberg, T. Story, Topological crystalline insulator states in $Pb_{1-x}Sn_xSe$. *Nat. Mater.* **11**, 1023–1027 (2012).

15. B. M. Wojek, P. Dziawa, B. J. Kowalski, A. Szczerbakow, A. M. Black-Schaffer, M. H. Berntsen, T. Balasubramanian, T. Story, O. Tjernberg, Band inversion and the topological phase transition in (Pb,Sn)Se. *Phys. Rev. B* **90**, 161202 (2014).

16. A. J. Strauss, Inversion of conduction and valence bands in $Pb_{1-x}Sn_xSe$ alloys. *Phys. Rev.* **157**, 608–611 (1967).

17. A. Okazaki, I. Ueda, The crystal structure of stannous selenide SnSe. *J. Physical Soc. Japan* **11**, 470 (1956).

18. L.-D. Zhao, S.-H. Lo, Y. Zhang, H. Sun, G. Tan, C. Uher, C. Wolverton, V. P. Dravid, M. G. Kanatzidis, Ultralow thermal conductivity and high thermoelectric figure of merit in SnSe crystals. *Nature* **508**, 373–377 (2014).

19. N. K. Abrikosov, V. F. Bankina, L. V. Poretskaya, L. E. Shelimova, E. V. Skudnova, *Semiconducting II–VI, IV–VI, and V–VI Compounds* (Plenum Press, 1969).

20. R. Car, G. Ciucci, L. Quartapelle, Electronic band structure of SnSe. *Phys. Status Solidi B* **86**, 471–478 (1978).

21. M. Parenteau, C. Carlone, Influence of temperature and pressure on the electronic transitions in SnS and SnSe semiconductors. *Phys. Rev. B* **41**, 5227–5234 (1990).

22. A. Agarwal, Synthesis of laminar SnSe crystals by a chemical vapour transport technique. *J. Cryst. Growth* **183**, 347–351 (1998).

23. B. Subramanian, C. Sanjeeviraja, M. Jayachandran, Brush plating of tin(II) selenide thin films. *J. Cryst. Growth* **234**, 421–426 (2002).

24. H. Maier, D. R. Daniel, SnSe single crystals: Sublimation growth, deviation from stoichiometry and electrical properties. *J. Electron. Mater.* **6**, 693–704 (1977).

25. A. J. Patel, A. R. Jani, B. B. Nariya, A. K. Dasadia, M. K. Bhayani, Electrical transport properties of SnS and SnSe single crystals grown by direct vapour transport technique. *Chalcogenide Lett.* **6**, 549–554 (2009).

26. W. Zhang, H. S. Jeong, S. A. Song, Martensitic transformation in $Ge_2Sb_2Te_5$ alloy. *Adv. Eng. Mater.* **10**, 67–72 (2008).

27. S. Mori, S. Hatayama, Y. Shuang, D. Ando, Y. Sutou, Reversible displacive transformation in MnTe polymorphic semiconductor. *Nat. Commun.* **11**, 85 (2020).

28. Y. K. Lee, K. Ahn, J. Cha, C. Zhou, H. S. Kim, G. Choi, S. I. Chae, J.-H. Park, S.-P. Cho, S. H. Park, Y.-E. Sung, W. B. Lee, T. Hyeon, I. Chung, Enhancing p-type thermoelectric performances of polycrystalline SnSe via tuning phase transition temperature. *J. Am. Chem. Soc.* **139**, 10887–10896 (2017).

29. T. Inoue, H. Hiramatsu, H. Hosono, T. Kamiya, Nonequilibrium rock-salt-type Pb-doped SnSe with high carrier mobilities ≈ 300 cm^2/(Vs). *Chem. Mater.* **28**, 2278–2286 (2016).

30. J. C. Woolley, O. Berolo, Phase studies of the $Pb_{1-x}Sn_xSe$ alloys. *Mater. Res. Bull.* **3**, 445–450 (1968).

31. A. A. Volykhov, V. I. Shtanov, L. V. Yashina, Phase relations between germanium, tin, and lead chalcogenides in pseudobinary systems containing orthorhombic phases. *Inorg. Mater.* **44**, 345–356 (2008).

32. Y. Ikuhara, P. Pirouz, High resolution transmission electron microscopy studies of metal/ceramics interfaces. *Microsc. Res. Tech.* **40**, 206–241 (1998).

33. Y. Huang, C. Wang, X. Chen, D. Zhou, J. Du, S. Wang, L. Ning, First-principles study on intrinsic defects of SnSe. *RSC Adv.* **7**, 27612–27618 (2017).

34. Y.-R. Luo, *Comprehensive Handbook of Chemical Bond Energies* (CRC Press, 2007).

35. K. Kobayashi, A. Nakao, S. Maki, J. Yamaura, T. Katase, H. Sato, H. Sagayama, R. Kumai, Y. Kuramoto, Y. Murakami, H. Hiramatsu, H. Hosono, Structure determination in thin film

$Ba_{1-x}La_xFe_2As_2$: Relation between the $FeAs_4$ geometry and superconductivity. *Phys. Rev. B* **96**, 125116 (2017).

36. G. K. H. Madsen, J. Carrete, M. J. Verstraete, BoltzTraP2, a program for interpolating band structures and calculating semi-classical transport coefficients. *Comput. Phys. Commun.* **231**, 140–145 (2018).

37. J. Liu, T. H. Hsieh, P. Wei, W. Duan, J. Moodera, L. Fu, Spin-filtered edge states with an electrically tunable gap in a two-dimensional topological crystalline insulator. *Nat. Mater.* **13**, 178–183 (2014).

38. C. Zhang, Y. Liu, X. Yuan, W. Wang, S. Liang, F. Xiu, Highly tunable berry phase and ambipolar field effect in topological crystalline insulator $Pb_{1-x}Sn_xSe$. *Nano Lett.* **15**, 2161–2167 (2015).

39. P. S. Mandal, G. Springholz, V. V. Volobuev, O. Caha, A. Varykhalov, E. Golias, G. Bauer, O. Rader, J. Sanchez-Bárriga, Topological quantum phase transition from mirror to time reversal symmetry protected topological insulator. *Nat. Commun.* **8**, 968 (2017).

40. Y. Wang, G. Luo, J. Kiu, R. Sankar, N.-L. Wang, F. Chou, L. Fu, Z. Li, Observation of ultrahigh mobility surface states in a topological crystalline insulator by infrared spectroscopy. *Nat. Commun.* **8**, 366 (2017).

41. G. Pizzi, V. Vitale, R. Arita, S. Blügel, F. Freimuth, G. Géranton, M. Gibertini, D. Gresch, C. Johnson, T. Koretsune, J. Ibañez-Azpiroz, H. Lee, J.-M. Lihm, D. Marchand, A. Marrazzo, Y. Mokrousov, J. I. Mustafa, Y. Nohara, Y. Nomura, L. Paulatto, S. Poncé, T. Ponweiser, J. Qiao, F. Thöle, S. S. Tsirkin, M. Wierzbowska, N. Marzari, D. Vanderbilt, I. Souza, A. A. Mostofi, J. R. Yates, Wannier90 as a community code: New features and applications. *J. Phys. Cond. Matt.* **32**, 165902 (2020).

42. D. K. Hohnke, S. W. Kaiser, Epitaxial PbSe and $Pb_{1-x}Sn_xSe$: Growth and electrical properties. *J. Appl. Phys.* **45**, 892–897 (1974).
